# RETRACTED: Aldakheel et al. Green Synthesized Silver Nanoparticles Loaded in Polysaccharide Hydrogel Applied to Chronic Wound Healing in Mice Models. *Gels* 2023, *9*, 646

**DOI:** 10.3390/gels12080721

**Published:** 2026-08-14

**Authors:** Fahad M. Aldakheel, Dalia Mohsen, Marwa M. El Sayed, Mohammed H. Fagir, Dalia K. El Dein

**Affiliations:** 1 Department of Clinical Laboratory Sciences, College of Applied Medical Sciences, King Saud University, Riyadh 11433, Saudi Arabia; faldakheel@ksu.edu.sa; 2 Clinical Laboratory Sciences Program, Inaya Medical College, Riyadh 12211, Saudi Arabia; husseinfagir@inaya.edu.sa (M.H.F.); dkmohammed@inaya.edu.sa (D.K.E.D.); 3 Microbiology Department, National Research Centre, Giza 12622, Egypt; 4 Chemical Engineering and Pilot Plant Department, National Research Centre, Giza 12622, Egypt; dr.marwameid@gmail.com

The journal retracts the article titled “Green Synthesized Silver Nanoparticles Loaded in Polysaccharide Hydrogel Applied to Chronic Wound Healing in Mice Models” [1], cited above.

Following publication, concerns were brought to the attention of the Editorial Office regarding the integrity of an image and the underlying data supporting this publication [1]. In accordance with the journal’s procedures, the Editorial Office and the Editorial Board conducted an investigation, with input from the Research Integrity team.

During the investigation, it was determined that Figure 4 overlaps with material available from a commercial supplier’s website, without appropriate attribution or disclosure. Although the authors cooperated with the investigation, they were unable to provide sufficient documentation to clarify the origin and provenance of the published image or to satisfactorily resolve the original concerns.

As a consequence, the Editorial Board decided to retract this publication in accordance with MDPI’s retraction policy.

This retraction was approved by the Editors-in-Chief of the journal *Gels*.

The authors have been informed of this retraction and have indicated their disagreement with this decision.
